# Correction

**DOI:** 10.1111/pbi.14417

**Published:** 2024-06-24

Correction to ‘Overexpression of *Arabidopsis* and Rice stress genes' inducible transcription factor confers drought and salinity tolerance to rice’

K. Datta, N. Baisakh, M. Ganguly, S. Krishnan, K. Yamaguchi Shinozai, S.K. Datta, “Overexpression of *Arabidopsis* and Rice stress genes' inducible transcription factor confers drought and salinity tolerance to rice,” Plant Biotechnology Journal 10, no. 5 (2012): 579–586.

Figure 6 has been removed due to an inconsistency with Line B having one missing lane and band similarity to Line C.

We apologize for this inadvertent error.

